# Carer coping and resident agitation as predictors of quality of life in care home residents living with dementia: Managing Agitation and Raising Quality of Life (MARQUE) English national care home prospective cohort study

**DOI:** 10.1002/gps.4994

**Published:** 2018-10-01

**Authors:** Anne Laybourne, Gill Livingston, Sian Cousins, Penny Rapaport, Kate Lambe, Francesca La Frenais, Hannah Savage, Monica Manela, Aisling Stringer, Louise Marston, Julie Barber, Claudia Cooper

**Affiliations:** ^1^ UCL Division of Psychiatry London UK; ^2^ Camden and Islington NHS Foundation Trust, Services for Ageing and Mental Health, St. Pancras Hospital London UK; ^3^ Department of Primary Care and Population Health UCL London UK; ^4^ PRIMENT Clinical Trials Unit, UCL London UK; ^5^ UCL Department of Statistical Science London UK

**Keywords:** agitation, care homes, dementia, quality of life

## Abstract

**Objectives:**

The objectives of the study are (1) to test our primary hypothesis that carers using more dysfunctional coping strategies predict lower quality of life in care home residents living with dementia, and this is moderated by levels of resident agitation, and (2) to explore relationships between carer dysfunctional coping strategy use, agitation, quality of life, and resident survival.

**Methods:**

In the largest prospective cohort to date, we interviewed carers from 97 care home units (baseline, 4, 8, 12, 16 months) about quality of life (DEMQOL‐Proxy) and agitation (Cohen‐Mansfield Agitation Inventory) of 1483 residents living with dementia. At baseline, we interviewed 1566 carers about coping strategies (Brief COPE), averaging scores across care home units.

**Results:**

Carer dysfunctional coping strategies did not predict resident quality of life over 16 months (0.03, 95% CI −0.40 to 0.46). Lower resident quality of life was longitudinally associated with worse Cohen‐Mansfield Agitation Inventory score (−0.25, 95% CI −0.26 to −0.23). Survival was not associated with carer dysfunctional coping, resident quality of life, or agitation scores.

**Conclusions:**

Carer dysfunctional coping did not predict resident quality of life. Levels of resident agitation were consistently high and related to lower quality of life, over 16 months. Lack of association between carer dysfunctional coping and resident quality of life may reflect the influence of the care home or an insensitivity of aggregated coping strategy scores. The lack of relationship with survival indicates that agitation is not explained mainly by illness. Scalable interventions to reduce agitation in care home residents living with dementia are urgently needed.

**Key points**

- A third of people living with dementia experience symptoms of agitation, such as restlessness, pacing, shouting, and verbal or physical aggression, which is associated with poorer quality of life in care home residents living with dementia in England.
- Carer dysfunctional coping strategies may negatively impact upon quality of life of residents, perhaps most prominently among residents experiencing more symptoms of agitation as they would require carers to use more coping strategies and thus be more vulnerable to dysfunctional strategies.
- Based on study results, lower resident quality of life was longitudinally associated with greater agitation and carers' dysfunctional coping strategies did not predict quality of life over 16 months.
- Scalable interventions to reduce agitation in care home residents living with dementia are urgently needed.

## 1 INTRODUCTION

A third of people living with dementia experience symptoms of agitation, such as restlessness, pacing, shouting, and verbal or physical aggression^1^ These are more common in people living with moderate and severe compared to mild dementia.^2^ People living with dementia who are agitated are more likely to move to a care home, and family carer burnout can mediate this relationship.^3^ Fewer than half of nursing home residents report good quality of life,^4^ but there is a dearth of robust research about what enables or interferes with living well with dementia, or cost‐effective ways to maintain and improve quality of life in this setting.^5^, ^6^ Reflecting the difficulties experienced by family carers precipitating care home admission, within the care home, carers also report difficulties caring for people with agitation.^3^ In this article, “carer” means an employed member of a care home team.

Agitation may be 1 determinant of poorer quality of life for people living with dementia in care homes,^2^ because symptoms can make delivering care very challenging.^3^ This association is likely to be driven by impairments in the abilities of care home residents with dementia to communicate and the needs that they have which are unmet, as well as dementia‐related neurodegenerative changes.^7^ The Needs‐Driven, Dementia‐Compromised Behaviour theory posits that in dementia, problem behaviours arise from unmet needs or goals, including emotional (communication, comfort, physical contact), recreational (stimulation, including touch, music; enjoyable activities), or physical needs (eg, pain relief, thirst, hunger).^8^ People living with dementia may not know or be able to communicate their wishes. When carers are unavailable, unaware, or inadequately skilled in communicating, a lack of understanding or attendance to these individual needs may increase agitation.

Carers are likely to cope with the stress of caring for people living with dementia in different ways, with differing impacts on residents. In family carers of people living with dementia at home, dysfunctional coping such as avoidance, behavioural disengagement, or venting^9^ was found to mediate the relationship between their reported burden and use of potentially abusive behaviours.^10^ We therefore hypothesised that carers' dysfunctional coping strategies may similarly impact upon quality of life of residents. We expected a stronger relationship among residents who experienced more symptoms of agitation as they would require carers to use more coping strategies and thus be more vulnerable to dysfunctional strategies.

We previously reported cross‐sectional, baseline data from the Managing Agitation and Raising Quality of Life (MARQUE) research programme's naturalistic cohort study where agitation was associated with poorer quality of life in care home residents living with dementia in England.^2^ Currently, we report the longitudinal findings from this 16‐month cohort study. Our primary hypothesis was that carers using more dysfunctional coping strategies predicted lower resident quality of life, moderated by levels of resident agitation. To consider an alternative model that agitation is mainly or wholly explained by greater physical and cognitive illness severity, we tested our hypothesis that higher levels of agitation predicts decreased survival.

## 2 METHODS

Ethical approval was received from the National Research Ethics Committee London‐Harrow 14/LO/0034 (06/03/14).

### 2.1 Setting and sampling

Care homes across England were recruited through third sector partners, NHS trusts and clinicians, Care England, the NIHR Clinical Research Network, and the Enabling Research in Care Homes network. To ensure external validity and generalisability, our sampling frame comprised each provider type (voluntary, state, and private) and care provision (nursing, residential). We defined care home clusters as units within care homes with distinct care teams, managers, and activity schedules. If carers in units cross‐covered each other, we defined this as 1 cluster. The use of the term “cluster” in this study denotes a care home unit and is the unit of analysis.

### 2.2 Procedure

Care home managers agreed to the unit or home taking part in the study. Managers identified residents with a known clinical diagnosis of dementia using care home records. For all residents without a known dementia diagnosis, the Noticeable Problems Checklist (NPC)^11^ was completed by a member of the care team. This is a 6‐item validated questionnaire covering memory, basic self‐care, orientation, naming familiar people, and ability to follow conversations. Eligible participants were all residents with an existing dementia diagnosis or NPC score >2.

Carers asked eligible residents judged to have capacity to consent to the study if researchers could approach them. Willing residents were approached by research assistants who assessed their decisional capacity and, if appropriate, followed informed consent procedures to invite residents to consent into the study. With all other residents, the care home contacted the next of kin, asking if researchers could contact them. As per the Mental Capacity Act (2005), the next of kin was invited to act as a personal consultee to make a decision about research participation on behalf of the resident. If there was no appropriate personal consultee, a professional consultee was sought.

Individuals named as next of kin agreeable to research contact were asked to consent to providing personal demographic information and information about how frequently they visit the resident. They were invited to complete a proxy measure of resident quality of life.

Care home managers also provided a staff list, and permanent carers providing hands‐on care were invited to complete measures about coping, burnout, and care practices. Bank carers were eligible to participate if they exclusively worked in that care home or cluster. All carers gave written informed consent.

Care home managers identified appropriate members of the care team to complete proxy measures about consented residents' dementia severity, agitation, and quality of life.

All participants were recruited between 13 January 2014 and 12 November 2015.

### 2.3 Measures

Trained research assistants interviewed carers and residents at the care home. At baseline, researchers met with relatives at their preferred venue, usually the care home, their own home, or the research office. Interviews were carried out at baseline, 4, 8, 12, and 16 months, except for carer self‐report measures and care home‐level measures, which were captured at baseline only. Relatives completed follow‐up measures with a research assistant via telephone or face to face.

#### 2.3.1 Care home‐level measures

We recorded information about whether the care home provided personal care, nursing care, or both, and whether it was dementia‐registered or a dementia specialist home.

#### 2.3.2 Resident measures

We recorded demographic details and information about the use of prescribed medications over the previous 28 days. The following measures were administered:

1. Quality of life: The DEMQOL and DEMQOL‐Proxy are responsive, valid, and reliable measures of quality of life in people living with dementia. The DEMQOL‐Proxy is a 31‐item interviewer‐administered questionnaire answered by a professional or family carer. The score range is 31 to 124. The people with dementia who were able to were asked to complete the DEMQOL, a 28‐item interviewer‐administered questionnaire. Scoring range for this instrument is 28 to 121. As the DEMQOL has fewer questions than the DEMQOL‐Proxy, the totals are not directly comparable.^12^, ^13^ Higher scores indicate better quality of life.
2. Agitation: Agitation was measured using the Cohen‐Mansfield Agitation Inventory (CMAI), a 29‐item questionnaire with construct validity and interrater and test‐retest reliability to measure agitation in people with dementia in care homes.^14^, ^15^ The CMAI is an informant questionnaire, and each item scores from 1 to 7, with 1 meaning “never” and 7 “several times per hour.” The score sums individual items and ranges from 29 to 203. A score of >45 is usually regarded as clinically significant agitation.^16^
3. Dementia severity: Staff gave information so the researcher could rate the severity of dementia using the Clinical Dementia Rating. This is a reliable and valid instrument for rating severity of dementia.^17^ It is used to rate performance in memory, orientation, judgment and problem solving, community affairs, home and hobbies, and personal care, and this information was used to classify dementia severity into very mild, mild, moderate, or severe. An adaptation of the Clinical Dementia Rating was used; research assistants did not undergo the Washington University online training. However, they followed the structured grid during the interview.

#### 2.3.3 Staff self‐reported measures

Staff measures were at baseline only. All consenting carers completed the Brief Coping Orientations to Problems Experienced (Brief COPE) measure. This is a multidimensional coping inventory widely used to assess the different ways in which people manage in response to stress.^18^ Coping styles appear to be fairly constant over time.^19^ It is a self‐report questionnaire,^9^ and participants score each strategy from 1 (not doing it at all) to 4 (doing it a lot). These have been grouped into 3 larger subscales that show adequate psychometric properties in dementia family carers^19^: problem‐focussed (active coping, instrumental support and planning), emotion‐focussed (acceptance, emotional support, humour, positive reframing, and religion), and dysfunctional coping (behavioural disengagement, denial, self‐distraction, self‐blame, substance use, and venting). Mean values per care home cluster were calculated.

#### 2.3.4 Family carer measures

We recorded family carers' demographics and asked them to complete a proxy measure of quality of life (DEMQOL‐Proxy^13^) and tell us how often they visited the person with dementia.

## 3 ANALYSIS

Data were analysed using Stata version 14. Mixed effects linear regression models were used to examine the relationship between quality of life scores over 16 months and baseline dysfunctional coping level within the care home cluster. Three level models were used to allow for the repeated measurements over time and clustering by care home cluster. Interaction terms were included in the models to consider differential effects by CMAI agitation score. Models were initially unadjusted. Fully adjusted models included time, sex, age, dementia severity, marital status, and visits by the main family carer. Assumptions of fitted models were investigated. Because of the severely skewed distribution of CMAI scores, all models were refitted using a dichotomous measurement representing CMAI caseness (CMAI case defined using cutoff score >45). Models were also refitted using the family carer proxy‐rated DEMQOL score in place of carer proxy‐rated score.

Associations between time to death and coping, agitation, and quality of life were analysed using Cox proportional hazards models with shared frailties to account for clustering by care home cluster. Unadjusted and adjusted models were fitted. Adjustments were made for resident age, sex, dementia severity, antipsychotic use, marital status, number of times a month the family carer visited the resident, and the number of British National Formulary subchapters the residents' prescribed medication encompassed (representing a measure of resident's physical comorbidity (19)).

### 3.1 Sample size justification

In a previous trial, the correlation between dysfunctional coping in family carers and the quality of life of the person with dementia was −0.31.^20^ To detect this magnitude of correlation with 90% power and 5% significance requires 105 people living with dementia.^21^ Adjusting for clustering by care team (estimated average team size: 40 people living with dementia, intracluster correlation (0.075),^22^ impact of confounding (variance inflation factor = 2),^23^ and an expected average 2.5 repeated measurements/person (based on 30% dropout/year) and correlation between repeated quality of life measurements of 0.75 (from START trial data (20)) required a total sample size of 700. This number was inflated to 2800 to allow investigation of the interaction between coping strategy and high and low agitation groups.

During the study, it became apparent that the average cluster size would be less than 40. With a smaller cluster size and more clusters, fewer people living with dementia are required to maintain power at 90%. Recalculations indicate that with 15 per cluster, the overall target would be 1466 people living with dementia (requiring 97 clusters). This was achieved in the study, and extra power gained by an increase in the number of repeated measures per resident (3.6 rather than 2.5 originally anticipated).

## 4 RESULTS

### 4.1 Recruitment and retention

We contacted 114 care homes, and 75% (n = 86) agreed to participate. Seven homes were subdivided into >1 cluster, totalling 18 clusters. The sample at baseline, therefore, was 97 clusters. Of these, 39 provided personal care, 13 nursing care, and 45 nursing and personal care. There were 86 care home units registered as providing dementia care, and 42 as providing dementia‐specialist care.

The total number of eligible care home residents was 3035 (86.2%); of these, 2825 (93.1%) were approached by researchers (directly or through a proxy); 1489 (52.7%) consented to participation. The common reasons for nonparticipation were refusal (27.3%) and the care team being unable to contact the family consultee to ask permission for researchers to contact them (17.6%). Three hundred (20.1%) had capacity to consent to the study at baseline, and we used consultees for the remainder; 1281 (86.0%) had a pre‐existing clinical diagnosis of dementia, and the remainder scored >2 on the NPC. There were 6 consented residents who died before data were collected, so analyses at baseline are based on 1483. The number of recruited residents per cluster ranged from 2 to 55 (median 14). One thousand eighty‐one (86.0%) of consenting residents had an identified family member who agreed to participate. One thousand five hundred sixty‐six carers completed the measure of coping. Numbers of carers per cluster ranged from 3 to 54 (median 15).

**Figure 1:**
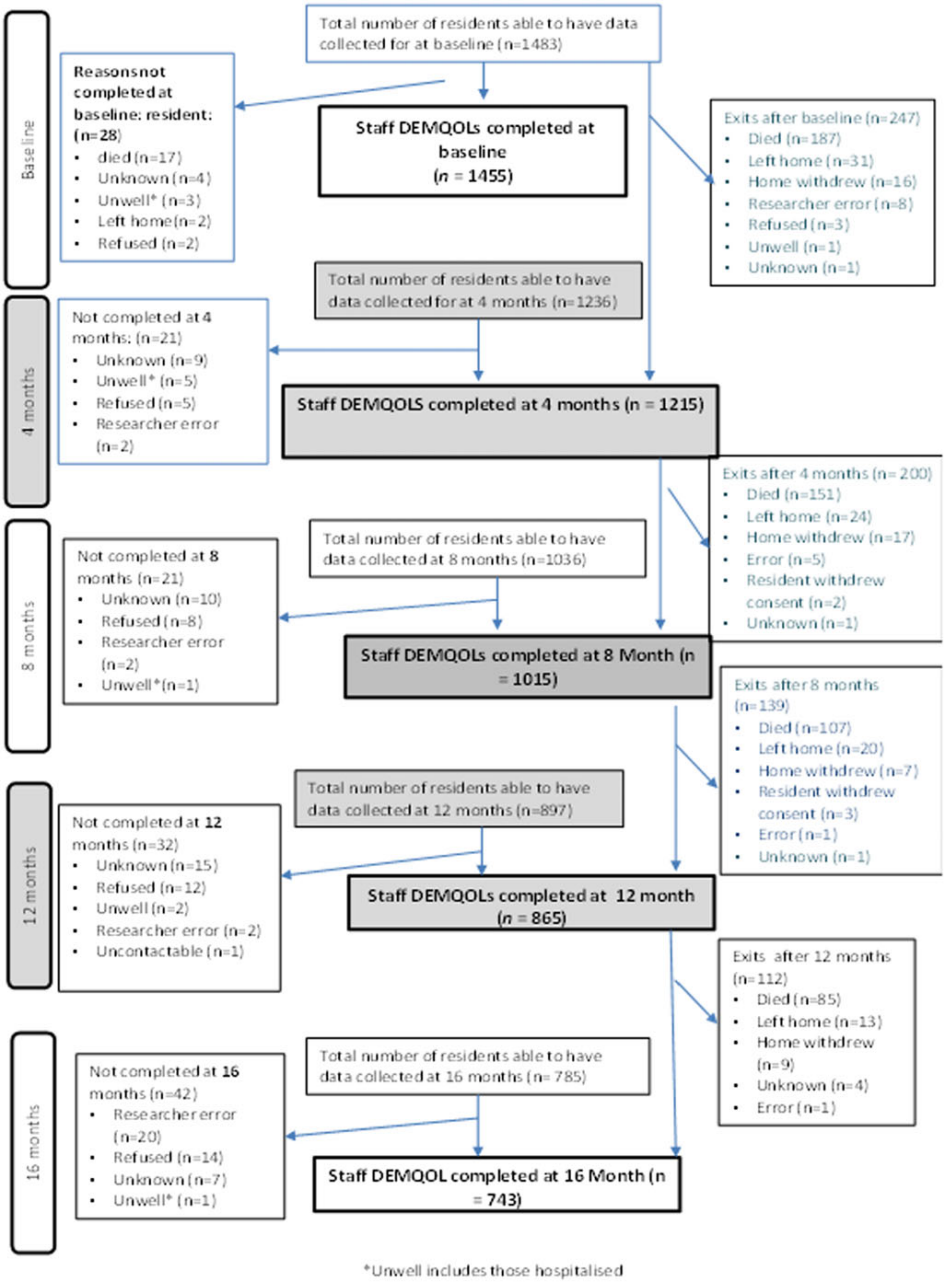
Flow diagram for completion of primary outcomes (carer‐rated DEMQOL) [Colour figure can be viewed at wileyonlinelibrary.com]

Our flow diagram (Figure 1) shows resident recruitment to and retention in the study. Median follow‐up time was 1.34 years (interquartile range (IQR): 0.68 to 1.44). Four care homes withdrew from the study after baseline; in 2 cases, this was because the home closed; 1 unit withdrew because the study was perceived as too time consuming and 1 unit did not give a reason.

### 4.2 Sample description

The majority of participants were female, widowed, and 71% were living with moderate to severe dementia (Table 1). They took a median of 7 medications, and had a mean age of 85 years. They received a median of 6 visits a month from the family carer who completed their DEMQOL‐Proxy. At baseline, the median (IQR) for paid carer dysfunctional coping scores was 16 (13 to 20; N = 1566). Aggregated at care home level, the median score was 17 (IQR 16 to 18; N = 97). Agitation and quality of life scores remained fairly stable and changed little over time (Table 2).

**Table 1:** Characteristics, dementia severity, agitation scores, quality of life, medication, and antipsychotic use of participating residents at baseline

Variable	N	Frequency (%) Unless Stated Otherwise
Female	1483	1026 (69%)
Age	1437	Mean (SD): 85 (9)
Family visits from main carer per month	1243	Median (IQR): 6 (3, 13)
Marital status	1424	
Married/common law		345 (24%)
Single, separated, divorced		287 (20%)
Widowed		792 (56%)
Dementia severity	1458	
Mild or very mild		427 (29%)
Moderate		482 (33%)
Severe		549 (38%)
Number of medications taken	1483	Median (IQR): 7 (5, 10)
Antipsychotic use	1483	248 (17%)
CMAI	1424	Median (IQR): 41 (33, 55)
CMAI >45	1424	569 (40%)
DEMQOL staff proxy	1455	Median (IQR): 104 (95, 110)
DEMQOL family carer proxy	1054	Median (IQR): 101 (90, 109)

Abbreviations: SD, standard deviation; IQR, interquartile range.

**Table 2:** CMAI and DEMQOL scores over time

	Median (IQR) Unless Stated Otherwise				
	Baseline	4 months	8 months	12 months	16 months
CMAI	41 (33, 55) N = 1424	39 (32, 54) N = 1201	40 (32, 53) N = 999	39 (32, 55) N = 851	39 (31, 52) N = 737
CMAI >45: frequency (%)	569 (40%) N = 1424	460 (38%) N = 1201	367 (37%) N = 999	320 (38%) N = 851	260 (35%) N = 737
DEMQOL staff proxy	104 (95, 110) N = 1455	105 (94, 111) N = 1215	106 (97, 111) N = 1015	106 (98, 111) N = 865	106 (99, 11) N = 743
DEMQOL family carer proxy	101 (90, 109) N = 1054	103 (92, 109) N = 699	103 (93, 1009) N = 536	102 (94, 109) N = 414	104 (93, 109) N = 318

Abbreviation: IQR, interquartile range.

### 4.3 Relationship of dysfunctional coping and CMAI scores to quality of life

Contrary to our hypothesis, there was no evidence of an association between resident quality of life over 16 months and carer baseline dysfunctional coping scores (0.06, 95% CI −0.39 to 0.52; N = 1457) or even after adjusting for sex, age, dementia severity, marital status, and visits by the main family carer (0.03, 95% CI −0.40 to 0.46; N = 1174). Carer proxy‐rated DEMQOL scores over 16 months were associated with CMAI scores over 16 months, indicating lower quality of life scores for those with worse agitation. This association was evident in unadjusted analyses (coefficient −0.25, 95% confidence interval (CI) −0.27 to −0.23; N = 1450) and in the fully adjusted model that included dysfunctional coping strategy use and the other potential confounders (−0.25, 95% CI −0.26 to −0.23; N = 1174). Dysfunctional coping was not a significant predictor in this model. The model extended to include an agitation (CMAI) by dysfunctional coping interaction term showed no evidence that the relationship between quality of life score over 16 months and coping was changed by level of agitation (*P* = 0.147). Alternative models where CMAI caseness was used in place of CMAI score gave similar conclusions (CMAI case cutoff >45 score). Cohen‐Mansfield Agitation Inventory caseness over 16 months was associated with lower quality of life scores over 16 months (adjusted difference in means −6.96, 95% CI −7.70, −6.23; N = 1174), but there was no evidence of an interaction with dysfunctional coping (*P* = 0.269). In models that used family carer proxy‐rated DEMQOL score in place of carer proxy‐rated score, results were again very similar: CMAI score predicted quality of life over 16 months in a fully adjusted model (−0.06, 95% CI −0.08 to −0.03; N = 994), and dysfunctional coping use score did not (0.35, 95% CI −0.16 to 0.85; N = 994).

### 4.4 Predictors of survival

Five hundred eight of 1470 participants died during the study period; median time to death was 7.4 months (interquartile range 4.2 to 11.6). In models adjusted for age, sex, dementia severity, number of medications, and whether taking antipsychotics (N = 1146) including CMAI caseness (score of 46+), neither CMAI caseness hazard ratio (HR 0.80, 95% CI 0.64‐1.01), baseline staff proxy DEMQOL score (HR 1.00, 1.00 to 1.01), nor baseline dysfunctional coping score (HR 1.00, 0.94 to 1.08) were significant predictors of survival (Table 3). When we repeated analyses using CMAI score instead of caseness, results were very similar. As expected, those living with mild or moderate dementia, younger residents, and women survived longer. Taking antipsychotic medication or number of medication classes were not significant predictors of survival.

**Table 3:** Fully adjusted (N = 1146) survival models

	Hazard Ratio	95% Confidence Interval
Baseline CMAI 46+	0.800	0.635, 1.007
Baseline staff proxy DEMQOL	0.999	0.990, 1.009
Baseline dysfunctional coping	1.003	0.936, 1.076
Resident age	1.056	1.040, 1.072
Sex		
Male	Ref.	
Female	0.636	0.502, 0.806
Dementia severity		
Mild	0.500	0.377, 0.663
Moderate	0.656	0.516, 0.834
Severe	Ref.	
Number of medications taken	1.009	0.980, 1.039
Antipsychotic use	0.998	0.747, 1.333
Marital status		
Married/common law	Ref.	
Single, separated, divorced	1.262	0.889, 1.791
Widowed	1.014	0.756, 1.361
Family visits from main carer per month	1.011	0.999, 1.023

## 5 DISCUSSION

In this well‐powered, naturalistic care home study, the largest to date, we did not demonstrate our primary hypothesis that greater use of dysfunctional coping strategies by carers would predict lower resident quality of life or greater levels of agitation. Levels of agitation and quality of life of residents living with dementia, rated by paid carers, remained fairly constant over the 16‐month follow‐up period. Higher levels of resident agitation predicted lower quality of life. These longitudinal findings build on those reporting this association in cross‐sectional data.^2^ We hypothesised that higher levels of agitation would predict survival, but this was not supported by these data.

We hypothesised that being cared for by carers who use dysfunctional care strategies would lower resident quality of life, in particular, among residents who had symptoms of agitation when they were first recruited and therefore require more carer coping. Although it seems logical that caring practices will impact on quality of life, we did not demonstrate our hypothesis. Carers cope with caring challenges within a set of multilevel systems that determine how care is delivered and therefore residents experience life. For homes that are part of a bigger chain, there is a wider macro system of the provider organisation; for all homes, there is a meso system of the care home and a microsystem of a shift or team influences. While most care homes intend to deliver person‐centred care, in reality, care work is often task‐focussed and stressful^24^ with a discrepancy between how people would like to care and the reality of what they feel able to achieve.^25^ Home policies can limit the individual coping strategies that are permissible within that system. There may be linguistic barriers too.^26^

Availability and accessibility of pleasant, meaningful activities for residents with dementia may be important in preventing agitation, and we did not measure the activities individual residents engaged in. We reported from MARQUE baseline analyses that the overall environment, good staffing levels, and overall time spent in activities in a particular home were not associated with agitation or quality of life.^2^ We did not measure carer burden, and we do not know whether paid carers who reported using dysfunctional coping strategies were talking about this in reaction to the stresses of caring, to organisational issues (such as poor pay or conditions), or to other life stresses.

An alternative explanation for not demonstrating our primary hypothesis is that the coping strategies carers reported in the Brief COPE did not sufficiently capture the interpersonal dynamics between residents and carers when someone became agitated. While the Brief COPE is a valid measure of general coping style, our study design could not capture how a carer's prevailing style of coping was influenced and modified by different caring situations and care recipients. Future research may benefit from integrating more in‐depth observational measures that capture this. A further explanation could be that carers may be reluctant to report dysfunctional coping, or may not carry out care in line with their self‐perceived care practices in an environment which they do not control. The Brief COPE has been validated and used widely with family carers of people with dementia^19^ but less so with paid carers, although it has been used across a range of populations including nurses.^27^ It is possible that residents or their families who refused participation or who could not be contacted were more agitated or had more severe dementia. We approached carers while they were at work and providing care, so perhaps those who struggled to cope were more likely to refuse.

In adjusted models, neither agitation nor quality of life predicted survival. This may be because those who are less agitated enter care homes at a later stage of dementia and an older age and thus survived less long. This would also explain our findings that neither antipsychotics nor number of medication classes predicted survival once age, sex, and dementia stage were known. The lack of relationship with survival indicates that agitation is not mainly explained by illness.

The lack of a link between quality of life and agitation and home environment, carer coping, physical illness, or survival may indicate that agitation is complex and an end point with complex aetiologies. The Needs‐Based Dementia‐Compromised Behaviour model will not be the only story; there is increasing evidence that symptoms such as agitation are caused by structural deficits in neural networks. A recent synthetic review suggests structural and functional deficits in brain regions associated with emotional regulation and salience.^28^ Agitation may therefore arise from a reduced capacity to regulate emotional responses and/or attentional resources, and possibly reduced problem‐solving ability. It could be that fear or anxiety or misunderstanding others' actions drives agitation, and future work should consider multiple theoretical perspectives.

We provide here the most comprehensive social evidence to date of a longitudinal link between agitation and quality of life for care home residents living with dementia. Residents' significant levels of agitation did not reduce over time. This may be because although they became more familiar with the care home, they also have a more severe level of dementia. It is unsurprising that living with agitation for periods of months and years negatively impacts quality of life. Effective, scalable care home interventions to reduce agitation and promote quality of life are thus needed and important. Within the MARQUE programme, we have developed and are evaluating a care team intervention to reduce agitation and improve quality of life of residents living with dementia.

## FUNDING STATEMENT

Economic and Social Research Council (ESRC) and National Institute for Health Research (NIHR): ES/L001780/1. CC and GL are supported by the UCLH NIHR Biomedical Research Centre. CC, PR, and GL were partly supported by the National Institute for Health Research (NIHR) Collaboration for Leadership in Applied Health Research and Care (CLAHRC) North Thames. The views expressed are those of the author(s) and not necessarily those of the NHS, the NIHR, or the Department of Health.

## CONFLICT OF INTEREST

None declared.

## DATA ACCESSIBILITY STATEMENT

Data will be made available on the UCL Research Data Repository (http://www.ucl.ac.uk/isd/services/research‐it/research‐data/repository).
